# Action Semantics at the Bottom of the Brain: Insights From Dysplastic Cerebellar Gangliocytoma

**DOI:** 10.3389/fpsyg.2018.01194

**Published:** 2018-07-12

**Authors:** Sabrina Cervetto, Sofía Abrevaya, Miguel Martorell Caro, Giselle Kozono, Edinson Muñoz, Jesica Ferrari, Lucas Sedeño, Agustín Ibáñez, Adolfo M. García

**Affiliations:** ^1^Laboratory of Experimental Psychology and Neuroscience, Institute of Cognitive and Translational Neuroscience, INECO Foundation, Favaloro University, Buenos Aires, Argentina; ^2^Departamento de Educación Física y Salud, Instituto Superior de Educación Física, Universidad de la República, Montevideo, Uruguay; ^3^National Scientific and Technical Research Council, Buenos Aires, Argentina; ^4^Departamento de Lingüística y Literatura, Facultad de Humanidades, Universidad de Santiago de Chile, Santiago, Chile; ^5^Neuropsychiatry Department, Institute of Cognitive Neurology, Buenos Aires, Argentina; ^6^Universidad Autónoma del Caribe, Barranquilla, Colombia; ^7^Center for Social and Cognitive Neuroscience, School of Psychology, Universidad Adolfo Ibáñez, Santiago de Chile, Chile; ^8^Centre of Excellence in Cognition and its Disorders, Australian Research Council (ARC), Sydney, NSW, Australia; ^9^Faculty of Education, National University of Cuyo, Mendoza, Argentina

**Keywords:** cerebellar atrophy, neurodegeneration, action pictures, object pictures, embodied cognition

## Abstract

Recent embodied cognition research shows that access to action verbs in shallow-processing tasks becomes selectively compromised upon atrophy of the cerebellum, a critical motor region. Here we assessed whether cerebellar damage also disturbs explicit semantic processing of action pictures and its integration with ongoing motor responses. We evaluated a cognitively preserved 33-year-old man with severe dysplastic cerebellar gangliocytoma (Lhermitte-Duclos disease), encompassing most of the right cerebellum and the posterior part of the left cerebellum. The patient and eight healthy controls completed two semantic association tasks (involving pictures of objects and actions, respectively) that required motor responses. Accuracy results via Crawford’s modified *t*-tests revealed that the patient was selectively impaired in action association. Moreover, reaction-time analysis through Crawford’s Revised Standardized Difference Test showed that, while processing of action concepts involved slower manual responses in controls, no such effect was observed in the patient, suggesting that motor-semantic integration dynamics may be compromised following cerebellar damage. Notably, a Bayesian Test for a Deficit allowing for Covariates revealed that these patterns remained after covarying for executive performance, indicating that they were not secondary to extra-linguistic impairments. Taken together, our results extend incipient findings on the embodied functions of the cerebellum, offering unprecedented evidence of its crucial role in processing non-verbal action meanings and integrating them with concomitant movements. These findings illuminate the relatively unexplored semantic functions of this region while calling for extensions of motor cognition models.

## Introduction

Motor brain networks play critical roles in grounding action meanings (verbal and non-verbal concepts related to bodily motion) and integrating them with ongoing manual movements (Buccino et al., 2005; Ibáñez et al., 2013; García and Ibáñez, 2014, 2016a). This association has been repeatedly shown for cortical regions (e.g., the primary and supplementary motor cortices) (Pulvermüller, 2005; Fischer and Zwaan, 2008; Vukovic et al., 2017) and frontostriatal networks affected by movement disorders (Cardona et al., 2013; García and Ibáñez, 2014; Kargieman et al., 2014; Melloni et al., 2015; Birba et al., 2017a). However, the contributions of the cerebellum, another key motor hub, have received comparatively lesser attention (García et al., 2016a; Guell et al., 2017). To bridge this gap, here we report the first assessment of explicit action-semantic processing and motor-semantic integration in a rare patient with bilateral cerebellar damage due to a dysplastic gangliocytoma (Lhermitte-Duclos disease).

The two domains in question are selectively or differentially compromised in patients with movement disorders (Kotz et al., 2009; Cardona et al., 2013). In Parkinson’s and Huntington’s disease, atrophy of frontostriatal motor loops impairs implicit and explicit processing of words (Fernandino et al., 2013a; Bocanegra et al., 2015), sentences (Fernandino et al., 2013b; Cardona et al., 2014), and images (Ibáñez et al., 2013; Bocanegra et al., 2015; García et al., 2017b,d) evoking bodily motion, while also disrupting predictable interference or facilitation effects (García and Ibáñez, 2016a) that such stimuli produce on concomitant hand movements (Ibáñez et al., 2013; Cardona et al., 2014; Kargieman et al., 2014; Melloni et al., 2015; Buccino et al., 2018). Notably, such deficits emerge irrespective of the patients’ domain-general impairments (Ibáñez et al., 2013; Bocanegra et al., 2015, 2017; García et al., 2017b) and even in preclinical disease stages (Kargieman et al., 2014; García et al., 2017d). It follows that damage to regions implicated in motor function can markedly disrupt the embodiment of action semantics.

While the above evidence comes from frontostriatal lesion models, the same could be hypothesized regarding the cerebellum, another hub critically involved in motor control and motor learning (Fabbro, 2000; Ramnani, 2006; Glickstein and Doron, 2008). Indeed, this region has been associated with the construction of internal models, or simulations (Ito, 1993, 2008; Ramnani, 2006), and it has been argued to play critical roles in embodied cognitive evolution (Barton, 2012). More particularly, cerebellar atrophy in genetic ataxia has been associated with selective action-verb impairments in lexical decision, a task that does not require explicit semantic processing (García et al., 2016a; Guell et al., 2017).

The latter finding, in particular, suggests that the cerebellum may be critical for grounding action meanings in shallow-processing verbal tasks – i.e., when semantic access is unnecessary for task completion. However, no study has assessed whether cerebellar damage also disturbs explicit action-semantic processing in non-verbal tasks, let alone its integration with ongoing motor responses. Thus, it remains unknown whether the distinct role of the cerebellum in motor semantics holds across processing modalities (verbal vs. pictorial) and levels of semantic access (implicit vs. explicit). To bridge this gap, we assessed a patient with severe dysplastic cerebellar gangliocytoma and eight healthy controls on two picture-based semantic association tasks (one involving objects, the other involving actions) requiring hand responses. In line with previous results (García et al., 2016a) and current embodied models (García and Ibáñez, 2016a), we hypothesized that cerebellar damage could selectively impair both the semantic association of actions and motor-semantic integration. In sum, we aimed to profit from this unique case to gain new insights into the contributions of the cerebellum to embodied cognitive functions.

## Materials and Methods

### Case Description

Patient NA is a 35-year-old, Spanish-speaking, right-handed Argentine man with 14 years of formal education. The patient reported a family history of neurological disease (his grandfather had dementia), psychiatric disease (his grandmother suffered from depression), and an antecedent of sudden death (his older brother died 3 months after birth).

On December 20, 2015, at age 33, NA experienced vertigo, low pressure, and generalized body weakness. Four days later, he manifested progressive dysarthria. At the end of January 2016, he suffered from sudden loss of consciousness but resumed normal activities after a few days. Throughout the following month, persistent signs of dysarthria were accompanied by reduced right-hand agility and progressive gait instability – mainly due to right-leg abnormalities. FLAIR and T2 MRI sequences revealed mild hyperintensity on the cerebellum without contrast enhancement, alongside thickened folia, small cysts, and sparing of the fourth ventricle. A posterior biopsy, together with histological and immunohistochemical studies, confirmed the diagnosis of dysplastic cerebellar gangliocytoma (Lhermitte-Duclos disease) as WHO stage IV.

In March 2016, NA started pharmacological treatment, shifting between Valcas (250 mg qd), Logical (200 mg tid), and Gabapentin (100 and 200 mg tid). That same year, on September 27, NA was hospitalized after experiencing aggravated vertigo, oscillopsia, and ataxia. Motor-system impairment was variously documented. In addition to right-sided horizontal gaze nystagmus (grade 2) and hearing deficits (negative Rinne’s test on the right side and left-lateralized Weber’s test), neurological examination revealed mild dysarthria, loss of balance (positive Rhomberg’s test), right-dominant muscular hypotonia with preserved force, motor nerve disturbances (positive Hoffman’s test on the right side), and ataxic gait. A follow-up MRI revealed an expansive right cortical-subcortical cerebellar lesion, characterized by hypointensity in T1 and corresponding hyperintensity in T2 and FLAIR, weighted signals with pseudocystic formations and no contrast enhancement, perilesional edema with mass effect on adjacent structures and the fourth ventricle, a right cerebellar nodular mass corresponding to a primary neo-proliferative lesion, and a discrete intensity change on the left cerebellum. No other signs of atrophy or malformations were observed, and lesions were essentially restricted to cerebellar structures. Given the rarity of Lhermitte-Duclos disease – with roughly 220 cases reported by 2006 (Robinson and Cohen, 2006)–, alongside its highly focal compromise of the cerebellum and its pervasive impact on motor function (Marcus et al., 1996; Nowak and Trost, 2002), this case offers a unique opportunity to test our hypothesis.

### Control Participants

The patient’s performance on the experimental tasks was compared with that of eight right-handed healthy men with no history of neurological or psychiatric disease. This control group matched the patient in terms of age (31.6 ± 5.53, *p* = 0.82) and education level (15.6 ± 2.7, *p* = 0.58). The study was carried out in accordance with the recommendations of the Ethics Committee of the Institute of Cognitive Neurology (INECO, now a host institution of the Institute of Cognitive and Translational Neuroscience), with written informed consent from all subjects. All participants gave written informed consent in accordance with the Declaration of Helsinki, and written informed consent was obtained from patient NA for the publication of this case report. The protocol was approved by the Ethics Committee of INECO.

### Neuroimaging: Lesion Localization

**FIGURE 1:**
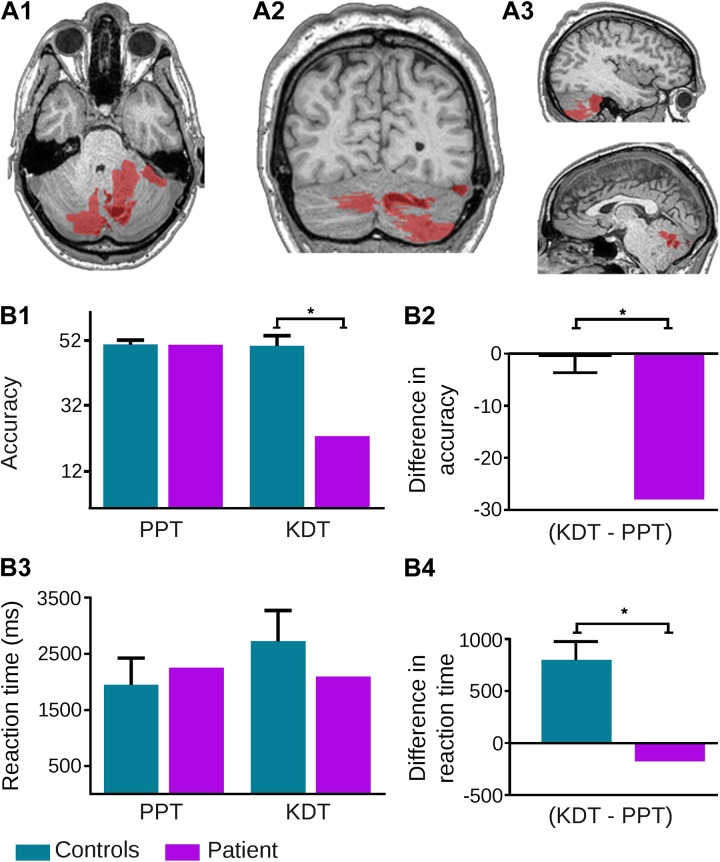
Neuroanatomical and behavioral evidence from patient NA. **(A)** Extent of the tumor highlighted in red on the original T1 MRI scan of the patient’s brain: **(A1)** axial view; **(A2)** coronal view; **(A3)** sagittal section – right (up) left (down). **(B)** Behavioral performance of patient and controls on the Pyramids and Palm Trees (PPT) test and the Kissing and Dancing Test (KDT). **(B1)** The patient showed significantly reduced accuracy on the KDT (*p* < 0.001) but not on the PPT test (*p* = 0.93). The patient’s deficit on the KDT remained after covarying for global scores on the Montreal Cognitive Assessment (MoCA), the INECO Frontal Screening (IFS) battery, and the State version of the State Trait Anxiety Inventory (STAI-S) – all *p*-values < 0.001. **(B2)** Subtraction of PPT from KDT accuracy scores in each subject showed a difference of 28 answers for the patient and an average difference of –0.3 in controls; this difference was significant (*p* = 0.001) and it remained so after covarying for MoCA (*p* = 0.005), IFS (*p* = 0.01), and STAI-S (*p* = 0.004) scores. **(B3)** Reaction times for the patient were similar to those of controls on both the PPT test (*p* = 0.59) and the KDT (*p* = 0.32). **(B4)** However, subtraction of reaction times on the PPT test from those of the KDT in each subject revealed notably longer latencies (∼800 ms more) for the latter in controls, and virtually null differences in the patient; such a difference between the patient and controls was significant (*p* = 0.005) and it remained so after covarying for MoCA (*p* < 0.001), IFS (*p* = 0.01), and STAI-S (*p* = 0.004) scores. The asterisk (^∗^) indicates significant differences.

Following previous procedures (García-Cordero et al., 2015, 2016; Melloni et al., 2016), an expert neurologist (JF) manually traced the patient’s lesion in native space. A T1 scan shows the extension of damage, comprising various parts of the cerebellum. Coordinates obtained from the Automated Anatomical Labeling software (Tzourio-Mazoyer et al., 2002) indicated that cerebellar damage included most of the right anterior and posterior hemisphere (comprising lobules VII, VIIB, VI, and IX, as well as crus I and II regions), part of the left posterior lobe (lobules VII, VIIB, IX, X, and crus II), and a few areas from the vermis (regions VII, VIII, IX, and X) (**Figure 1, panels A1–A3**).

### Instruments

#### Neuropsychological and Psychiatric Evaluation

The assessment protocol included instruments tapping overall cognitive status, executive functions, and anxiety levels.

The participants’ overall cognitive state was assessed with the Montreal Cognitive Assessment (MoCA), a sensitive tool for detecting cognitive dysfunction in populations with atrophy of motor regions, such as the basal ganglia (Gluhm et al., 2013; Bocanegra et al., 2015, 2017) and the cerebellum (Mercadillo et al., 2014; Dogan et al., 2016; García et al., 2016a). The MoCA covers eight cognitive domains, namely: visuospatial/executive skills, naming, memory, attention, language, abstraction, delayed recall, and orientation. It has a maximum of 30 points, and its total score is corrected for the participant’s years of education.

In addition, executive functions were assessed through the INECO Frontal Screening (IFS) battery (Torralva et al., 2009). This tool has proved sensitive for population with damage to motor regions (Cardona et al., 2014; Bocanegra et al., 2015; García et al., 2016a, 2017a). The IFS taps domains such as motor programming, conflict resolution, inhibitory control, and working memory. This battery comprises 20 items, and its maximum score is 30.

Anxiety levels during the cognitive tasks were assessed via the state version of the State Trait Anxiety Inventory (STAI-S; Spielberger et al., 1970). The psychometric properties of this instrument attest to its reliability and validity to detect situational anxiety (Spielberger et al., 1980) in the general population (Barnes et al., 2002). It has also been used in patients with damage in motor regions, such as the basal ganglia (Leritz et al., 2004; Tinaz et al., 2011) and the cerebellum (Zawacki et al., 2002; Orsi et al., 2011). The STAI-S comprises 20 self-report items inquiring on the examinee’s current feelings. Each item rates severity on a 1–4 scale, so that the total score ranges from 20 to 80.

#### Semantic Association of Objects and Actions

Semantic association of objects and actions was assessed through the Pyramids and Palm Trees (PPT) test (Howard and Patterson, 1992), and the Kissing and Dancing Test (KDT) (Bak and Hodges, 2003), respectively. In both tests, participants must choose which of two pictures is most closely related to a cue picture. Each test comprises 52 trials, yielding a maximum score of 52. These instruments have revealed specific deficits in patients with damage to motor regions, such as the basal ganglia (Cardona et al., 2014; Bocanegra et al., 2015; García et al., 2017b,d), and other less focused injuries that include cerebellar atrophy (Baez et al., 2013).

The patient and all controls were tested individually in a dimly illuminated room. They sat comfortably at a desk, in front of an Intel Core i5-3470 PC equipped with a monitor (Lenovo 15.6” 16:9 HD LED backlight display) and a QWERTY keyboard (gx gaming).

For this study we implemented a computerized version of both tasks, designed and run on DMDX software ^1^, as to automatically record accuracy and reaction-time (RT) data and thus assess the impact of action-semantic processing on concurrent manual actions (i.e., responses made by pressing of predefined keyboard buttons). In each trial, the cue picture was located at the top of the screen and the two option pictures appeared at the bottom, in a pyramid-like arrangement. All three images were presented simultaneously and they remained on the screen for 5 s. If no response was made within this time interval, a blank screen was shown for a maximum of 5 s before the next trial appeared.

Participants were instructed to respond as fast and accurately as possible by pressing the left or the right arrow of the keyboard with two fingers of the dominant hand, indicating their choice of the picture at the corresponding location. Each key press was logged to compute accuracy and RT, and it triggered a 1-s blank screen followed by the next trial. Prior to each task, the instructions were recapped on-screen and four additional items were presented for practice purposes. The patient performed the PPT test first and then the KDT, whereas both tasks were counterbalanced across control participants.

### Statistical Analysis

Demographic, neuropsychological, psychiatric, and behavioral data were compared between the patient and controls via two-tailed Crawford’s modified *t*-tests (Crawford and Howell, 1998; Crawford and Garthwaite, 2002), as done in previous studies (Couto et al., 2013a,b; Sedeño et al., 2014; García et al., 2016a,b; Birba et al., 2017b). This test allows comparing test scores from one or more individuals with norms derived from small samples. It has been proved to be robust for non-normal distributions and it presents low rates of type-I error (Crawford and Howell, 1998; Crawford and Garthwaite, 2002, 2012; Crawford et al., 2009, 2011).

Also, to assess performance differences between the PPT test and the KDT, RT data from the patient and the controls were analyzed via the Revised Standardized Difference Test (RSDT) (Crawford and Garthwaite, 2005). This test examines the difference between a subject’s scores on two tasks relative to the difference observed in the control group, considering the distribution of values in the latter and its correlation between tasks. As the modified *t*-test, the RSDT controls for the type-I error rate in the case of small control samples (Crawford and Garthwaite, 2005). In RT analyses, all trials exceeding 2 SDs from the subject’s mean were removed as outliers (less than 10% of the trials in all cases).

Finally, to determine whether hypothesized differences between the patient and controls were influenced by the former’s cognitive status, executive skills, or anxiety levels, all analyses were repeated with global MoCA, IFS, and STAI-S scores as independent covariates. These analyses were based on a Bayesian Test for a Deficit allowing for Covariates (BTD-Cov) when Crawford’s *t*-test was applied, and a Bayesian Standardized Difference Test allowing for Covariates (BSDT-Cov) when the RSDT was applied (Crawford et al., 2011). Effect sizes obtained from these methods are reported as point estimates (Z_CCC_ and Z_DCCC_ for covaried results from the modified *t*-test and RSDT, respectively), as suggested in a previous study (Crawford et al., 2010). In all analyses, alpha levels were set at *p* < 0.05.

## Results

### Cognitive Status, Executive Functions, and State Anxiety Level

No significant differences emerged between the patient and controls in the MoCA, the IFS battery, or the STAI-S (**Table 1**). Therefore, the patient showed no cognitive deficits or abnormal anxiety levels.

**Table 1:** Overall cognitive profile and anxiety levels.

	Patient	Controls (*n* = 8)	*p*-value	*t*-value	z_CC_
MoCA	26	27.13 (1.64)	0.54	−0.65	−0.69
IFS	17	24 (3.96)	0.14	−1.68	−1.77
STAI-S	35	33.25 (6.14)	0.78	0.27	0.29

*Standard deviations are indicated between parentheses. MoCA, Montreal Cognitive Assessment (Nasreddine et al., 2005); IFS, INECO Frontal Screening battery (Torralva et al., 2009); STAI-S, state version of the State Trait Anxiety Inventory (Spielberger et al., 1970). Statistical analyses were conducted with Crawford’s modified t-test (Crawford and Howell, 1998; Crawford and Garthwaite, 2002).*

### Semantic Performance: Accuracy Results

Compared to controls, the patient exhibited normal accuracy on the PPT test (*t* = −0.09, Z_CC_ = −0.09, *p* = 0.93), with a marked impairment on the KDT (*t* = −8.41, Z_CC_ = −8.94, *p* < 0.001). This differential pattern remained after covarying for MoCA (PPT: Z_CCC_ = −0.1, *p* = 0.94; KDT: Z_CCC_ = −10.18, *p* < 0.001), IFS (PPT: Z_CCC_ = −0.41, *p* = 0.78; KDT: Z_CCC_ = −8.84, *p* < 0.001). and STAI-S (PPT: Z_CCC_ = −0.19, *p* = 0.88; KDT: Z_CCC_ = −9.74, *p* < 0.001) scores (**Figure 1, panel B1**). Also, a comparison of between-task differences (KDT minus PPT) highlighted the markedly differential outcome for the patient in the KDT (*t* = 5.29, Z_DCC_ = 6.51, *p* = 0.001) and corroborated their independence from general cognitive skills (MoCA: Z_DCCC_ = 7.5, *p* = 0.005), executive functions (IFS: Z_DCCC_ = 6.44, *p* = 0.01), and state anxiety (STAI-S: Z_DCCC_ = 7.71, *p* = 0.004) (**Figure 1, panel B2**).

### Semantic Performance: RT Results

Moreover, although RTs revealed no significant differences between the patient and controls on either the PPT test (*t* = 0.58, Z_CC_ = 0.62, *p* = 0.59) or the KDT (*t* = −1.08, Z_CC_ = −1.15, *p* = 0.32) (**Figure 1, panel B3**), analysis of between-task differences via Crawford’s RSDT revealed a specific abnormality: whereas controls responded more slowly on the KDT than on the PPT test, no such difference was observed in the patient (*t* = 4.08, Z_DCC_ = 5.58, *p* = 0.005). This result, too, was uninfluenced by general cognitive skills (MoCA: Z_DCCC_ = 13.47, *p* < 0.001), executive functions (IFS: Z_DCCC_ = 6.32, *p* = 0.01), and state anxiety (STAI-S: Z_DCCC_ = 7.44, *p* = 0.004) (**Figure 1, panel B4**).

## Discussion

This is the first study examining explicit processing of action-related meanings and their integration with ongoing actions in a patient with extensive cerebellar damage. The patient exhibited a selective impairment of action semantics, relative to object semantics, together with a probable alteration of the predictable motor-semantic integration pattern observed in controls. Furthermore, both patterns remained after covarying for executive skills. Below we discuss these findings in turn, addressing their theoretical and clinical relevance.

### Cerebellar Damage and Action Semantics

First, the patient showed selective deficits in processing action (as opposed to object) semantics. This highlights the role of the cerebellum in grounding movement-related meanings, arguably because of its critical role in motor function (Ramnani, 2006; Manto et al., 2012). In healthy subjects, action semantics is differentially related to activity in the primary motor and premotor cortices (Jirak et al., 2010; Vigliocco et al., 2011; García and Ibáñez, 2016a). Moreover, this domain is selectively affected by damage to those regions (Neininger and Pulvermüller, 2003) or to frontostriatal motor loops (García et al., 2016b, 2017a,b; Birba et al., 2017a). Our results extend these findings, showing that damage to the cerebellum, another critical motor hub, can also lead to selective deficits in action-semantic processing.

A previous report on action-verb processing in cerebellar ataxia revealed selective deficits in this category, even though more demanding lexical classes, such as abstract verbs, were preserved (García et al., 2016a). Of note, the latter study found this impairment through a lexical decision task, involving implicit semantic access. Our study shows that cerebellar damage can lead to action semantic deficits even in explicit picture-based tasks, suggesting that the cerebellum plays a crucial role in grounding action-related meanings irrespective of presentation modality (verbal vs. pictorial) or mode of access (implicit vs. explicit), as previously observed for other motor regions (Pulvermüller, 2005; Jirak et al., 2010; Birba et al., 2017a). Taken together, this finding supports the view that the cerebellum may play a transmodal role in the embodiment of action-related meanings (García and Ibáñez, 2016a; Birba et al., 2017a), alongside more general contributions to semantic processing at large (Booth et al., 2007; De Smet et al., 2007; Murdoch, 2010; Barton, 2012; Mariën et al., 2014).

Compatibly, in fact, a feasible interpretation of our RT results is that the patient was also impaired in motor-semantic integration, another relevant embodied domain (García and Ibáñez, 2014, 2016a). In healthy subjects, processing of effector-specific action meanings can predictably interfere with contiguous hand movements (as indexed by increased RTs). This has been shown in multiple experimental settings, including semantic decision via single-key presses (e.g., Dalla Volta et al., 2014), semantic congruency judgment paradigms (e.g., Bernardis and Gentilucci, 2006; Barbieri et al., 2009), and keyboard-based verb-copying tasks (García and Ibáñez, 2016b). However, in disorders characterized by motor-network atrophy, such as Parkinson’s and Huntington’s disease, systematic motor-semantic integration effects are abolished (Ibáñez et al., 2013; Cardona et al., 2014; Kargieman et al., 2014; Buccino et al., 2018), in the context of abnormal task-specific neural signatures (Melloni et al., 2015), and even before the onset of overt motor symptomatology (Kargieman et al., 2014). For example, in these populations, contrary to healthy controls, congruency between response-hand shape and the hand-position denoted by action verbs fails to significantly modulate RTs (Ibáñez et al., 2013; Cardona et al., 2014; Kargieman et al., 2014). Likewise, healthy individuals respond more slowly to stimuli involving motor affordances (i.e., pictures and words depicting graspable, as opposed to non-graspable, objects), but no such selective delay is observed in PD patients (Buccino et al., 2018). As proposed by (García and Ibáñez, 2018), these findings would show that motor-network atrophy disturbs the integration of manual movements with processing of action-related stimuli, due to a disruption of embodied mechanisms.

In line with this claim and its supporting evidence, our study offers the first indication that similar patterns could emerge upon damage to the cerebellum. Indeed, whereas manual responses in controls were slower for action than for object stimuli, no such interference was observed in the patient. Given that the same cerebellar regions are engaged by the execution and the observation of actions (Gazzola and Keysers, 2009), we surmise that joint recruitment of motor and action-semantic processes led to a competition for resources in controls, while such natural integrative dynamics became disturbed upon cerebellar damage in the patient. In fact, motor-network disruptions have been shown to result in the recruitment of alternative non-motor pathways during processing of action-related stimuli (Abrevaya et al., 2017), which warrants the possibility that similar abnormal grounding effects could be triggered by cerebellar compromise. In this sense, the cerebellum seems critical not only for the processing of action semantics *per se*, but also for the integration of action meanings with ongoing motoric behavior.

Admittedly, although the results support the proposed interpretation, other factors could be contributing to the observed patterns. First, larger RTs in the KDT than the PPT test in controls could be partially driven by differential stimulus-related demands in each task. Although both instruments are similar in structure and overall difficulty (Bak and Hodges, 2003), their respective images are not matched for fine-grained variables which could impact behavioral outcomes, such as visual complexity, familiarity, or age of acquisition – for examples from normative picture-based studies, see (Cycowicz et al., 1997; Manoiloff et al., 2010). Therefore, subtle differences in such variables may have partly contributed to the behavioral differences observed in controls. Second, the patient’s pattern of more errors and faster RTs on the PPT test could also be influenced by a trade-off between speed and accuracy: should this task prove harder than the KDT, instruction-induced time pressure could have led the patient to respond more quickly than his actual processing speed requires, resulting in a greater propensity to errors. While these factors cannot be fully ruled out as partial contributors to the results, their impact is only speculative and could well run in parallel to (rather than in lieu of) the argued abolished action-interference effect, which has been previously reported in patients with motor-network damage (Buccino et al., 2018). Further research would be necessary to establish the relative role of these factors on the observed effects.

Of note, the two patterns of deficit exhibited by the patient emerged despite otherwise normal cognitive performance, and they survived after covariation with MoCA, IFS, and STAI-S scores. This indicates that both forms of embodied disturbances were not dependent on the patient’s overall cognitive status, executive functioning, or state anxiety levels. Similar patterns have been observed in previous studies showing distinctive action-semantic deficits in Parkinson’s (Bocanegra et al., 2015, 2017) and Huntington’s (García et al., 2017b) disease, indicating that such impairments were *sui generis* (i.e., not secondary to domain-general dysfunctions). By the same token, our study suggests that action-semantic and motor-semantic-integration difficulties may emerge as *primary* manifestations not only following early damage to frontostriatal motor networks, but also to posterior motor hubs (in particular, the bilateral cerebellum).

### Implications

Our work has theoretical and clinical implications. In the last decades, the cerebellum has been acknowledged as a key hub for adaptive control functions, including the modeling, prediction, and organization of motor, cognitive, and emotional behaviors (Schmahmann, 1991; Barton, 2012; Koziol et al., 2014; Guell et al., 2017). More particularly, sparse evidence has hinted to its role in semantic processing, through coarse-grained tasks such as word selection (Silveri and Misciagna, 2000; Murdoch, 2010) and story comprehension (Mar, 2011). However, as established in a recent consensus paper, the contributions of the cerebellum to semantic and other higher-order domains represent an “ongoing enigma” (Mariën et al., 2014). In light of our results, and considering current theoretical proposals (Barton, 2012), we propose that important theoretical breakthroughs can be made by studying cerebellar function from an embodied perspective.

Anticipatory control loops in the cerebellum have been implicated in the mental rehearsal and imagination of actions, as well as in the prediction of their distal and abstract consequences (Koziol et al., 2014). Among other things, these processes would support the simulation (Jeannerod, 2001; Hesslow, 2002) and emulation (Grush, 2004) of bodily states, as tacitly assumed by recent models characterizing the prediction and understanding of external events (Schubotz, 2007). These general findings broadly support the view that the cerebellum plays a distinct role in grounding action-specific meanings. However, no specific proposals have been advanced in such a direction, arguably because there is no consensus on the role of motor and non-motor cerebellar regions in processing action-related rules (Balsters and Ramnani, 2008; Koziol et al., 2014).

In light of our results, we propose that the cerebellum could constitute a key hub in the vast motor-preferential network supporting the embodiment of action meanings (García and Ibáñez, 2016a; García et al., 2016a). Notably, most neuroanatomical models of action semantics (Pulvermüller, 2005, 2018; Garagnani and Pulvermüller, 2016) emphasize the putative role of cortical motor regions, failing to acknowledge the contributions of subcortical and cerebellar motor hubs. This may largely be the case because several relevant studies have found cerebellar activity but failed to include it in their discussions or accompanying summary diagrams (Decety and Grèzes, 1999; Shapiro et al., 2005; Lauro et al., 2013), and because relevant lesion models of damage to subcortical motor regions have been overlooked in the field (Birba et al., 2017a). However, as shown by present results, previous evidence of action-language deficits following cerebellar atrophy (García et al., 2016a), and even some imaging studies of action semantics including results from the cerebellum (Saccuman et al., 2006; Rüschemeyer et al., 2007; Boulenger et al., 2009), the embodied foundations of action semantics may span across any and all regions subserving motor function.

This proposal also entails clinical implications. On the assumption that the contributions of the cerebellum to higher-order processes were restricted to managing novel situations, organizing responses or creating linguistic strategies (Copland et al., 2000), various authors have proposed that deficits triggered by cerebellar damage could be more accurately detected and characterized through assessments of complex domain-general processes (Murdoch, 2010). While such approaches are certainly useful, here we propose that more fine-grained examinations targeting specific semantic categories (in particular, those alluding to bodily motion) could represent a novel clinical alternative. Indeed, the very combination of tasks used in this study has revealed deficits in early (Bocanegra et al., 2015) and even preclinical (García et al., 2017b) stages of motor disorders characterized by subcortical motor-network atrophy.

Building on the notion that deficits in both action semantics and motor-language coupling could constitute sensitive biomarkers of frontostriatal motor loops (Ibáñez et al., 2013; García and Ibáñez, 2014; Birba et al., 2017a), our findings suggest that relevant tasks could also reveal subtle and primary signatures of cerebellar damage. In this sense, embodied semantic tasks could emerge as robust transdiagnostic tools for detecting early motor-network disruptions, irrespective of lesion site or etiology. However, replication and normative studies are needed to directly test this possibility.

## Limitations and Suggestions for Further Research

Two main limitations must be recognized in this work. First, the control sample had a modest size. However, our tests are considered robust with small control groups (∼5 subjects) (Crawford and Howell, 1998), as attested by previous single-case studies yielding robust findings with similar or even smaller control-sample sizes (Bak et al., 2006; García et al., 2016a, 2017c; Birba et al., 2017b), or even in the absence of control groups (Caramazza and Hillis, 1991; Silveri et al., 1998; Berndt and Haendiges, 2000). Second, our assessment was restricted to a single cognitive task, whereas the explored domains manifest in multiple ways. Notwithstanding, note that the KDT and the PPT test have shown good sensitivity for revealing differential and selective deficits in other pathologies (Bak and Hodges, 2004; Kargieman et al., 2014; Bocanegra et al., 2015; Tsermentseli et al., 2016; García et al., 2017b,d), which attests to their empirical relevance.

Looking forward, although single cases are crucial to determine potential links between cognitive impairments and neuroanatomical injuries (Dubois and Adolphs, 2016), future studies should test the replicability of our results in a broad population of cerebellar patients. In particular, regression models could be implemented between semantic performance and structural or functional neural correlates, with a view to identifying specific cerebellar regions implicated in action semantics. This could illuminate the controversy regarding the contributions of motor and non-motor portions of the cerebellum to processing of action-related information (Balsters and Ramnani, 2008; Koziol et al., 2014). Also, given that the cerebellum and the basal ganglia have profuse connections with classical language areas (Booth et al., 2007), assessment of structural and functional connectivity in patients with cerebellar damage could reveal the putative basis of potential embodied deficits, as revealed by relevant behavioral tasks. Finally, a comparison of action-semantic processing between cerebellar and non-cerebellar models of motor-network lesions could reveal informative dissociations and inform fine-grained models of embodied cognition.

## Conclusion

This study offers unprecedented evidence that cerebellar damage could alter explicit processing of action-related meanings and their integration with ongoing actions. These findings illuminate the relatively unexplored semantic functions of this region while calling for extensions of motor cognition models. Moreover, as previously shown in other movement disorders, embodied semantic tasks also offer promising alternatives for detecting early motor-network disruptions upon cerebellar damage. In this sense, the study of cerebellar contributions to action-semantic processing may afford a fruitful overarching framework for future basic and applied research in cognitive neuroscience.

## Author Contributions

AG, AI, and LS conceived the study. SA, MMC, and GK collected the data. SC, EM, JF, and AG analyzed the data. SA and SC designed **Figure 1**. SC and AG wrote the manuscript. AI and LS provided critical revisions on the successive drafts. All authors approved the manuscript in its final form.

## Conflict of Interest Statement

The authors declare that the research was conducted in the absence of any commercial or financial relationships that could be construed as a potential conflict of interest.

## Footnotes

1 http://www.u.arizona.edu/~kforster/dmdx/dmdx.htm
